# Guanethidine Restores Tetracycline Sensitivity in Multidrug-Resistant *Escherichia coli* Carrying *tetA* Gene

**DOI:** 10.3390/antibiotics13100973

**Published:** 2024-10-15

**Authors:** Xiaoou Zhao, Mengna Zhang, Zhendu Zhang, Lei Wang, Yu Wang, Lizai Liu, Duojia Wang, Xin Zhang, Luobing Zhao, Yunhui Zhao, Xiangshu Jin, Xiaoxiao Liu, Hongxia Ma

**Affiliations:** 1 College of Animal Science and Technology, Jilin Agricultural University, Xincheng Street No. 2888, Changchun 130118, China; zhaoxo4466@163.com (X.Z.); zhangmn2021@126.com (M.Z.); wang2311472521@163.com (Y.W.); 2 Institute of Animal Husbandry and Veterinary Medicine, Jilin Academy of Agricultural Science, Kemao Street No. 186, Gongzhuling 136100, China; zhangzhendu@126.com (Z.Z.); florawong1982@126.com (L.W.); 15662165476@163.com (L.L.); 15104487977@163.com (D.W.); 16624441717@163.com (X.Z.); a13630969537@163.com (L.Z.); yunhui0328@126.com (Y.Z.); 13894469000@163.com (X.J.); 3 College of Veterinary Medicine, Northwest A&F University, Xinong Street No. 22, Yangling 712100, China; 4 College of Life Sciences, Jilin Normal University, Haifeng Street No. 1301, Siping 136000, China; 5 The Engineering Research Center of Bioreactor and Pharmaceutical Development, Ministry of Education, Jilin Agricultural University, Xincheng Street No. 2888, Changchun 130118, China

**Keywords:** guanethidine, antibiotic adjuvant, tetracyclines, *tetA*, *Escherichia coli*

## Abstract

The worrying issue of antibiotic resistance in pathogenic bacteria is aggravated by the scarcity of novel therapeutic agents. Antibiotic adjuvants offer a promising solution due to their cost-effectiveness and high efficacy in addressing this issue, such as the β-lactamase inhibitor sulbactam (a β-lactam adjuvant) and the dihydrofolate reductase inhibitor trimethoprim (a sulfonamide adjuvant). This study aimed to discover potential adjuvants for tetracyclines from a list of previously approved drugs to restore susceptibility to *Escherichia coli* carrying the *tetA* gene. We have screened guanethidine, a compound from the Chinese pharmacopoeia, which effectively potentiates the activity of tetracyclines by reversing resistance in *tetA*-positive *Escherichia coli*, enhancing its antibacterial potency, and retarding the development of resistance. Guanethidine functions via the inhibition of the TetA efflux pump, thereby increasing the intracellular concentration of tetracyclines. Our findings suggest that guanethidine holds promise as an antibiotic adjuvant.

## 1. Introduction

The global proliferation of resistant pathogens is primarily attributed to the prevalence of antibiotic resistance [1]. Deaths caused directly or indirectly by antibiotic-resistant *E. coli* have always been have the most numerous among these pathogens [2]. However, the development of novel antibiotics targeting Gram-negative bacteria has been hindered by various obstacles such as high costs, lengthy cycles, low profitability, or the implementation of antibiotic restriction orders in certain countries [3]. Moreover, the unique membrane or cell wall structure of Gram-negative bacteria adds to the difficulty of discovering new antibiotics [4]. In contrast, antibiotic adjuvants offer a rapid, efficient, and cost-effective strategy that synergizes with antibiotics to enhance their activity or inhibit resistance [5,6]. Classic examples of antibiotic adjuvants still in clinical use today include the *β*-lactam inhibitor sulbactam (a *β*-lactam adjuvant) and the dihydrofolate reductase inhibitor trimethoprim (a sulfanilamide adjuvant) [7,8]. In recent years, researchers have increasingly focused on the potential of previously approved drugs (PADs) to discover new antibiotic adjuvants. For instance, the antidiabetic metformin has shown promise in restoring doxycycline susceptibility against multidrug-resistant bacteria [9]. Antibiotic adjuvants primarily target the following targets: enzyme inhibitors (such as beta-lactamase inhibitors), efflux pump inhibitors (such as 3,4-dibromopyrrole-2,5-dione, specializing in inhibiting the RND family efflux pump) [10], outer membrane permeabilizers (including cationic peptides SLAP-S25) [11], biofilm disruptors (such as peptide 1018) [12], and target enhancers (such as trimethoprim). However, research on antibiotic adjuvants targeting a specific resistance gene is relatively less.

Despite the prevalence of tetracycline resistance in bacteria nowadays, tetracyclines are widely used in animal husbandry due to their broad antibacterial spectrum, low price, few side effects, and growth-promoting properties [13,14,15]. However, it is worrying that tetracycline resistance is widespread in pathogenic bacteria, with resistance rates exceeding 80% and more than 30 types of tetracycline resistance genes identified [9,16,17,18]. The presence of the *tetA* gene is one of the most common types. The *tetA* gene encodes the tetracycline efflux pump protein TetA, which is resistant to all tetracyclines except tigecycline. TetA is a transmembrane protein that can recognize and bind to tetracycline antibiotics, and then use the proton motive force to pump these antibiotics out of the bacterial cell. In this way, TetA effectively lowers the antibiotic concentration inside the cell, so that the antibiotic cannot reach the concentration that inhibits bacterial growth and reproduction [19,20]. Recent research has uncovered how sensitive *E. coli* bacteria rapidly acquire resistance to tetracycline antibiotics by obtaining transferable plasmids carrying the *tetA* gene [21]. This finding indicates that pathogens that already had some level of resistance to tetracycline antibiotics are now developing even higher levels of resistance.

The guanidine group, which can be protonated in physiological environments, gains a positive charge and forms hydrogen bonds or electrostatic interactions with potential bacterial targets [22,23]. This group has attracted attention in the design and discovery of antibiotics. In this study, we screened potential tetracycline antibiotic adjuvants from PADs for their ability to reverse tetracycline resistance in *E. coli* strains carrying *tetA*. The PADs list involved in this study includes the Chinese Pharmacopoeia. In our screening of non-antimicrobial drugs containing guanidine in the Chinese Pharmacopoeia, we discovered guanethidine as a compound that effectively reverses tetracycline resistance in *E. coli* strains carrying *tetA*. Guanethidine enhances the antibacterial activity of tetracyclines and delays the development of resistance. The primary mechanism of guanethidine involves inhibiting the TetA efflux pump, which results in an increased intracellular accumulation of tetracyclines. Additionally, we investigated both the pharmacophoric group and in vitro effectiveness of guanethidine as an antibiotic adjuvant.

## 2. Results

### 2.1. Guanethidine Potentiates Tetracyclines against Multidrug-Resistant E. coli

**Figure 1:**
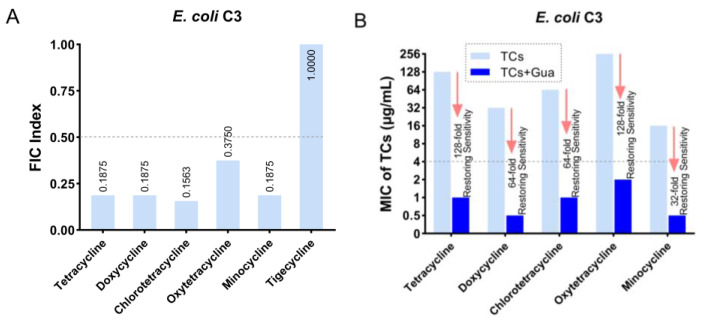
The antibacterial activity of guanethidine in combination with tetracyclines against *tetA*-positive *E. coli* C3. (**A**) The FIC index of guanethidine and tetracyclines. The FIC index (fractional inhibitory concentration index) is commonly used to define the interactions between two bioactive compounds. The FIC index is commonly used to assess the synergistic or antagonistic effects of antibiotics when used in combination with other antibiotics or antibiotic adjuvants against microorganisms. Synergy is defined as an FIC index of ≤0.5. (**B**) The MICs of tetracyclines with or without guanethidine (2.5 mg/mL), the fold of decrease in MICs, and whether sensitivity can be restored. MIC ≤ 4 μg/mL is sensitive, refer to CILS 2020. All experiments were conducted with four biological replicates.

The multidrug-resistant *E. coli* C3, which is *tetA* positive, is resistant to almost all tetracyclines except for tigecycline (the drug resistance pattern shown in Table S1), was selected as the type strain. The guanidine compound guanethidine was screened from the 2010 edition of the Chinese Pharmacopoeia II based on the chemical structure. It exhibited synergistic effects with almost all tetracyclines, except for tigecycline (Figure 1A), resulting in a 32-fold to 128-fold reduction of the minimum inhibitory concentration (MIC), restoring susceptibility to tetracyclines in resistant *E. coli* C3 (Figure 1B). However, guanethidine alone displayed weak direct antibacterial activity with an MIC ≥ 5 mg mL.

**Figure 2:**
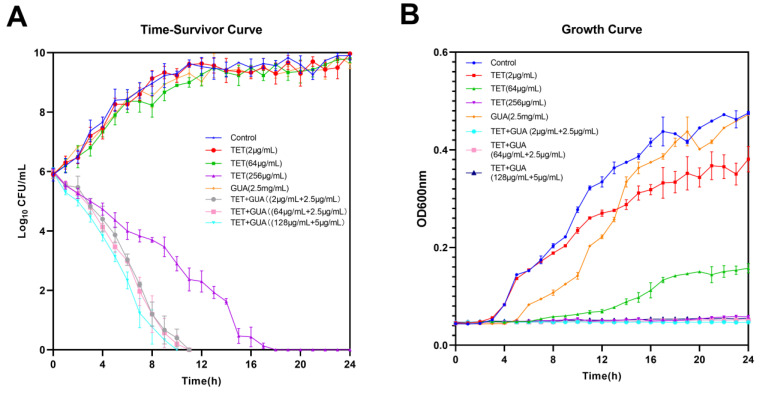
The antimicrobial efficiency of guanethidine in combination with tetracyclines against *E. coli* C3. (**A**) Time–Survivor Curves of guanethidine in combination with tetracyclines within 24 h. (**B**) Growth curves in 24 h. All experiments were conducted with three biological replicates.

To further elucidate the antimicrobial efficiency of guanethidine in combination with tetracyclines, we monitored bacterial growth and bactericidal kinetics within a 24 h period. Bacterial growth is completely suppressed and completely eradicated within 12 h. (Figure 2A,B).

**Figure 3:**
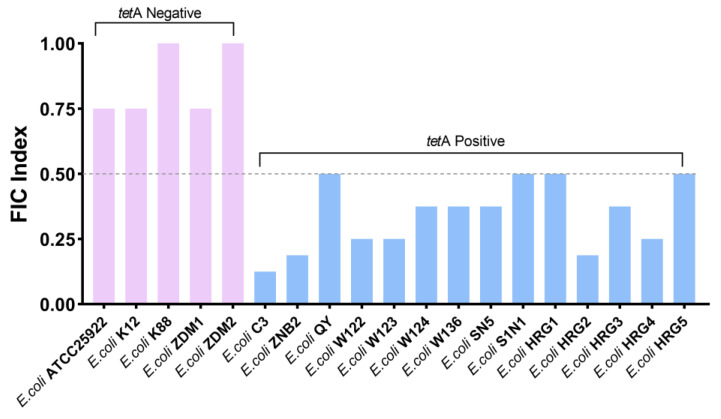
The FIC indices of guanethidine and tetracycline against various sources of *E. coli* with or without *tetA*. Synergy was defined as an FIC index of ≤0.5. All experiments were conducted with four biological replicates.

In addition, the synergistic effect of guanethidine and tetracycline was evaluated on fourteen strains of *E. coli* with *tetA* from various sources, as well as on five strains of *E. coli* without *tetA*. The results demonstrate that guanethidine and tetracycline exhibit synergistic activity against *E. coli* with *tetA*, while showing a minimal synergistic effect on *E. coli* without *tetA* (Figure 3).

**Figure 4:**
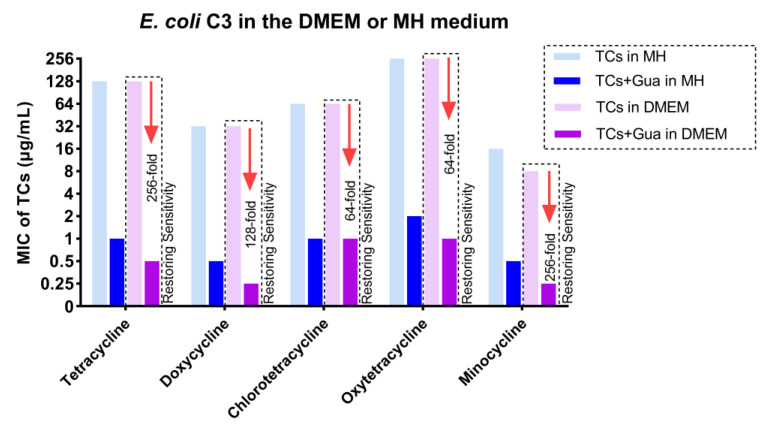
The MICs of tetracyclines against *E. coli* in the DMEM or MH medium, with or without guanethidine (2.5 mg/mL). MIC ≤ 4 μg/mL is sensitive, refer to CILS 2020. All experiments were conducted with four biological replicates.

To test the synergistic effect, a DMEM culture medium containing 1% serum similar to the in vivo environment was chosen, rather than the rich nutrient culture medium MHII typically used for rapid microbial growth. Guanethidine can still enhance tetracycline antibiotics and restore sensitivity to the multidrug-resistant strain *E. coli* C3 (Figure 4).

### 2.2. Guanethidine Retards the Development of Resistance in E. coli

**Figure 5:**
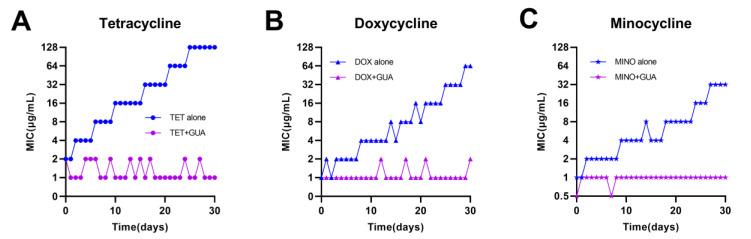
Emergence in *E. coli* ATCC25922 of resistance to tetracycline (**A**), doxycycline (**B**), and minocycline (**C**) with or without guanidine (2.5 mg/mL) after successive passages for 30 days.

The decline in antibiotic efficacy is primarily attributed to the rapid emergence of antibiotic resistance [24,25]. We evaluated the impact of guanethidine on the development of bacterial resistance to tetracycline antibiotics. In the presence of guanethidine, the MICs for tetracycline-sensitive *E. coli* ATCC 25922 only increased by up to 2-fold after continuous passage for 30 days in an environment with resistance to tetracycline, doxycycline, or minocycline. However, the MICs for *E. coli* ATCC 25922 increased by up to 64-fold after successive passages in a resistant environment with tetracycline, doxycycline, or minocycline alone. Notably, guanethidine effectively limited the development of tetracycline resistance in susceptible *E. coli* ATCC25922 (Figure 5A–C).

### 2.3. Guanethidine Affects the Integrity of the Outer Membrane

**Figure 6:**
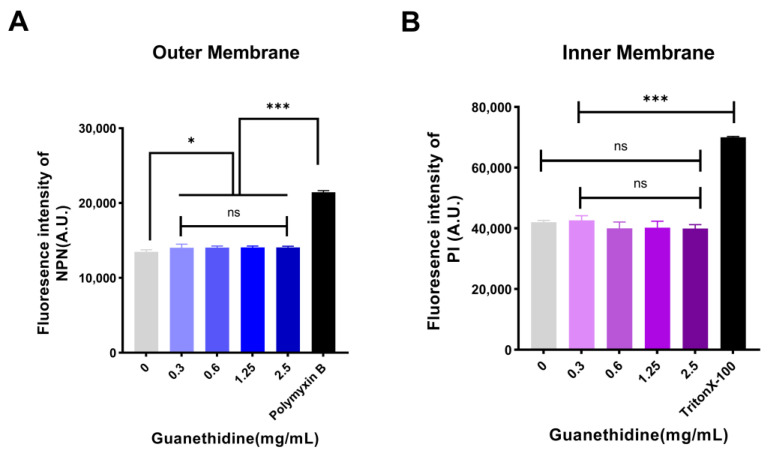
Effects of guanethidine (0–2.5 mg/mL) on the permeability of *E. coli* C3 cell membrane: (**A**) outer membrane and (**B**) inner membrane. The significance of the differences was analyzed by one-way ANOVA: ns, *p* > 0.05; *, *p* < 0.05; **, *p* < 0.01; and ***, *p* < 0.001. All experiments were performed with five biological replicates.

Some antibiotics disrupt cell membrane structure to kill bacteria [26,27]. NPN fluorescent probes were used to assess the integrity of the outer membrane, and PI fluorescent probes were used to detect the impact on the inner membrane. Compared to the blank control group, NPN fluorescence intensity decreased significantly, while PI fluorescence intensity showed no significant change, indicating that guanethidine affects the outer membrane integrity with minimal impact on the inner membrane (Figure 6).

### 2.4. Guanethidine Affects Bacterial PMF

**Figure 7:**
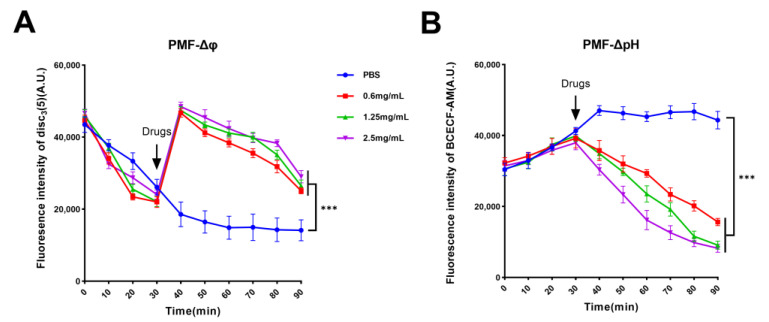
Effects of guanethidine (0–2.5 mg/mL) on the PMF of *E. coli* C3: (**A**) the transmembrane potential gradient (ΔΨ) and (**B**) pH gradient (ΔpH). The significance of the differences was analyzed by one-way ANOVA: ns, *p* > 0.05; *, *p* < 0.05; **, *p* < 0.01; and ***, *p* < 0.001. All experiments were performed with three biological replicates.

Guanethidine can affect the integrity of the bacterial cell membrane, which may indirectly impact the bacterial proton motive force (PMF) since the integrity of the cell membrane is crucial for maintaining a normal PMF [28]. Fluorescence probes DiSC_3_(5) and BCECF-AM were utilized to monitor changes in the transmembrane potential gradient (ΔΨ) and pH gradient (ΔpH) of PMF, respectively. The fluorescence remained stable after 30 min, and varying concentrations of guanethidine were added to measure the fluorescence intensity. Compared to the PBS control group, the fluorescence intensity of DiSC_3_(5) initially increased rapidly followed by a gradual decrease, indicating that guanethidine affects the bacterial membrane potential gradient (Figure 7A). Similarly, there was a significant decrease in fluorescence intensity observed with the BCECF-AM probe compared to the PBS control group, suggesting that guanethidine impacts the pH gradient (Figure 7B). Consequently, the addition of guanethidine influences the bacterial proton motive force.

**Figure 8:**
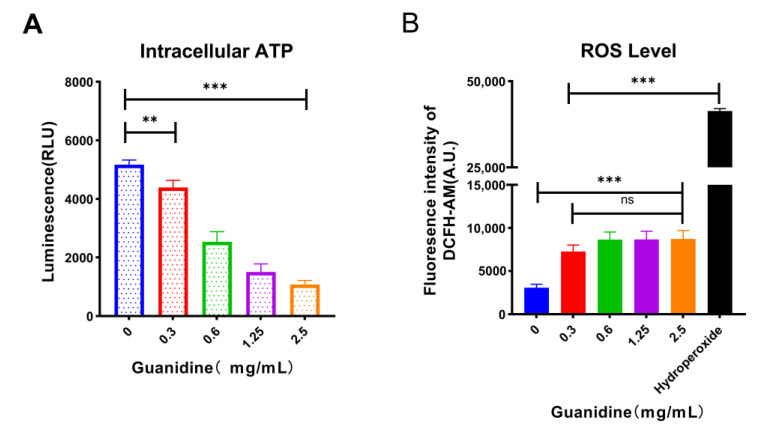
Effects of guanethidine (0–2.5 mg/mL) on the intracellular ATP and ROS of *E. coli* C3: (**A**) effects on intracellular ATP and (**B**) effects on intracellular ROS levels. The significance of the differences was analyzed by one-way ANOVA: ns, *p* > 0.05; *, *p* < 0.05; **, *p* < 0.01; and ***, *p* < 0.001. All experiments were performed with three biological replicates.

Subsequently, we measured intracellular ATP and ROS levels associated with the PMF. The intracellular ATP of *E. coli* C3 treated with guanethidine at various concentrations exhibited a significant decrease compared to the blank control, demonstrating a concentration-dependent decline (Figure 8A). Additionally, the addition of guanethidine at different concentrations stimulated an increase in intracellular ROS compared to the blank control group (Figure 8B).

### 2.5. Guanethidine Addition Is Correlated with an Enhanced Intracellular Concentration of Tetracycline in E. coli

**Figure 9:**
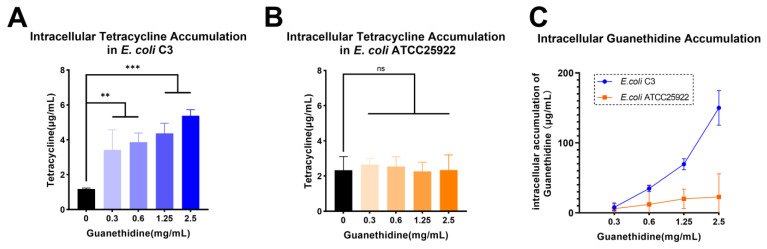
An increased intracellular accumulation of tetracycline in *E. coli* caused by guanethidine. (**A**) An intracellular accumulation of tetracycline with guanethidine (0–2.5 mg/mL) in *E. coli* C3. (**B**) An intracellular accumulation of tetracycline with guanethidine (0–2.5 mg/mL) in *E. coli* ATCC25922. (**C**) An intracellular accumulation of guanethidine in *E. coli* C3 or *E. coli* ATCC25922. The significance of the differences was analyzed by one-way ANOVA: ns, *p* > 0.05; *, *p* < 0.05; **, *p* < 0.01; and ***, *p* < 0.001. All experiments were performed with three biological replicates.

The ability of tetracyclines to inhibit bacterial protein synthesis is essential for their antibacterial activity [16], thus it is crucial to ensure sufficient drug accumulation within bacterial cells. LC-MS/MS was used to quantify the intracellular accumulations of tetracycline in *E. coli* C3 (with *tetA*) or *E. coli* ATCC25922 (without *tetA*) treated with different concentrations of guanethidine. The presence of guanethidine resulted in a concentration-dependent increase in intracellular tetracycline content against *E. coli* C3 (Figure 9A), while there was no significant change in the intracellular tetracycline accumulation for *E. coli* ATCC25922 (Figure 9B). Furthermore, the intracellular accumulation of guanethidine increased proportionally with its concentration (Figure 9C).

### 2.6. Guanethidine Did Not Significantly Affect the Expression Level nor the Integrity of the TetA Protein

**Figure 10:**
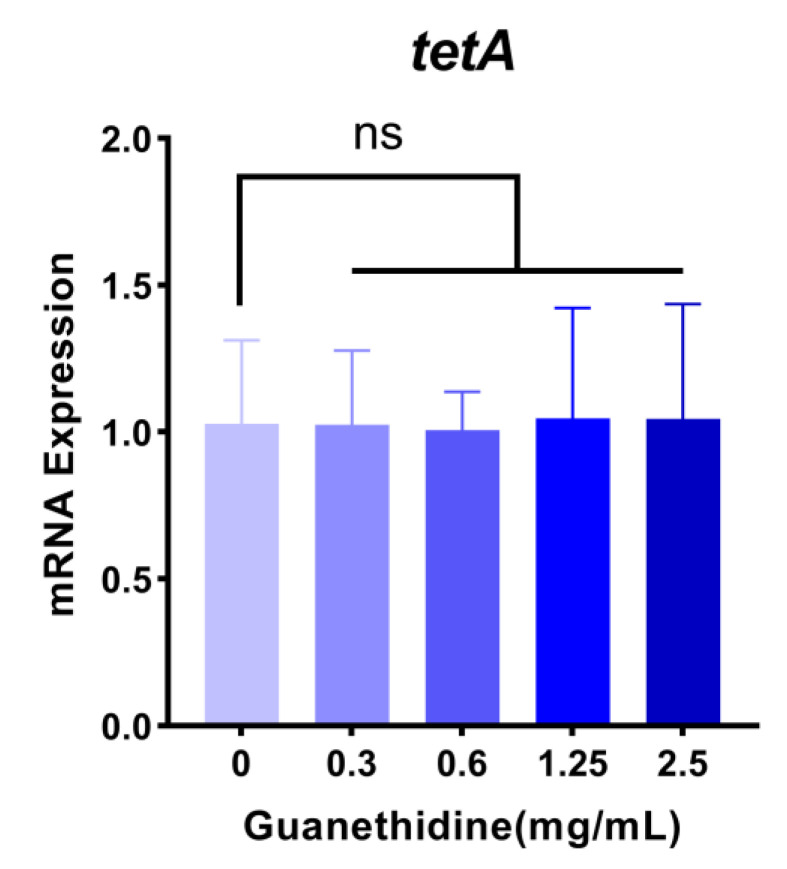
The effect of guanethidine (0–2.5 mg/mL) on the expression of the tetracycline-efflux pump gene *tetA*. The significance of the differences was analyzed by one-way ANOVA: ns, *p* > 0.05; *, *p* < 0.05; **, *p* < 0.01; and ***, *p*< 0.001. All experiments were performed with three biological replicates.

The expression of the *tetA* gene in *E. coli* C3 showed no significant changes following treatment with different concentrations of guanethidine, as revealed by RT-PCR analysis (Figure 10). In addition, the *tetA* gene in the *E. coli* C3 strain was analyzed through Sanger sequencing after being subjected to serial passaging for 7 d in the presence or absence of guanethidine. No nucleotide sequence breaks, losses, or mutations were observed compared to the untreated strain.

### 2.7. Guanethidine Inhibits the Activity of Efflux Pump Protein TetA

Guanethidine effectively enhances the activity of tetracycline antibiotics against *E. coli* strains carrying the *tetA* gene, which encodes a tetracycline efflux pump protein. This protein significantly compromises the efficacy of tetracycline by facilitating its extrusion from bacterial cells through the *tetA* efflux pump [20,29].

#### 2.7.1. Guanethidine Enhances the Effectiveness of Tetracycline against Genetically Engineered *E. coli* Expressing the *tetA* Gene

**Figure 11:**
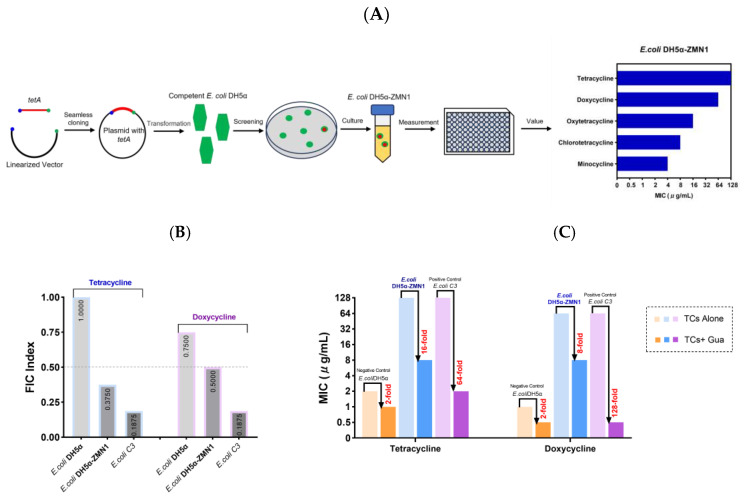
(**A**) The screening procedure of *E. coli* DH5α-ZMN1 (**B**) The FIC index of guanethidine and tetracyclines. Synergy is defined as an FIC index of ≤0.5. (**C**) The MICs of tetracyclines with or without guanethidine (2.5 mg/mL), folds decrease in MICs are shown in red. All experiments were performed with four biological replicates.

Using seamless cloning technology, we constructed a plasmid containing the *tetA* gene and transferred it into competent *E. coli* DH5α. Subsequently, we successfully isolated a strain named *E. coli* DH5α-ZmN1 which carries the *tetA* gene and demonstrates resistance to tetracyclines. The MICs of tetracyclines against *E. coli* DH5α-ZMN1 were determined to be up to 64 μg/mL (Figure 11A). Representative tetracycline and doxycycline were chosen to assess their combination with guanethidine. The combination exhibits a synergistic effect against genetically engineered *E. coli* DH5α-ZMN1, resulting in a 16-fold or 8-fold enhancement of tetracycline or doxycycline antibacterial activity, respectively (Figure 11B,C).

#### 2.7.2. The Molecular Docking Analysis Demonstrates the Stable Binding of Guanethidine to the TetA Pocket

**Figure 12:**
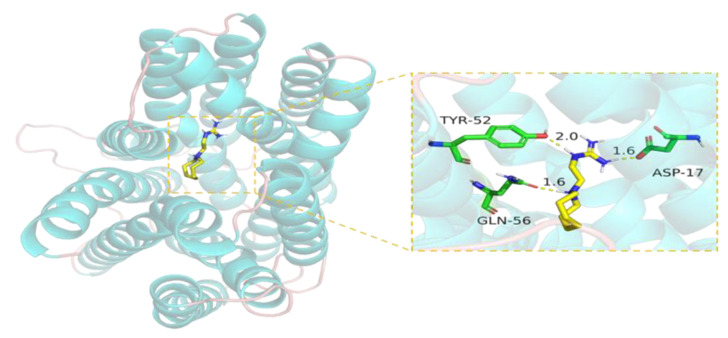
The predicted binding mode of protein TetA with the compound guanidine by molecular docking. The protein framework is tubular and stained bright blue, guanethidine is depicted in gray, and the yellow dashed line indicates hydrogen bond distances.

The binding capacity between guanidine and TetA, a specific efflux pump protein for tetracycline, was predicted by molecular docking. The binding energy of guanethidine to the protein TetA is −6.42 kcal/mol, which indicates a favorable interaction since the energy is below the threshold of −6 kcal/mol typically associated with a stable binding. It should be noted that a lower binding energy indicates a higher likelihood for the compound to bind to the protein. Guanethidine’s guanidine group can form multiple hydrogen bond interactions with active amino acid groups. According to the calculations made by MOE software (2020 version), guanethidine could bond with the active amino acid residues TYR-52, GLN-56, and ASP-17 in the TetA pocket (Figure 12). The average distance between the hydrogen bonds is 1.73 Å, which is within the typical range for effective hydrogen bonding (approximately 2.2 to 3.6 Å), indicating a strong binding force that significantly contributes to stabilizing the small molecule ligand. Therefore, guanethidine has the potential to stabilize the binding within the TetA protein’s pocket, thereby affecting the activity of the TetA protein.

### 2.8. The Guanidine Group Is the Crucial Group of the Synergistic Effect

**Figure 13:**
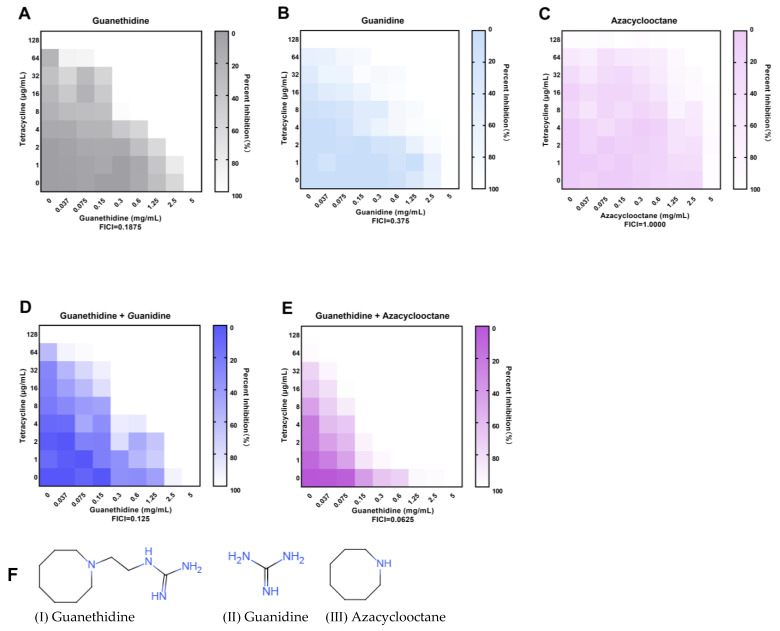
Checkerboard microdilution method to test the antimicrobial effect of tetracycline in combination with guanethidine and its derivatives. (**A**) The effect of tetracycline in combination with guanethidine. (**B**) The effect of tetracycline in combination with guanidine. (**C**) The effect of tetracycline in combination with azacyclooctane. (**D**) The effect of tetracycline in combination with guanethidine with the addition of additional subinhibitory concentrations of guanidine (1.25 mg/mL). (**E**) The effect of tetracycline in combination with guanethidine with the addition of additional subinhibitory concentrations of azacyclooctane (1.25 mg/mL). (**F**) The chemical formulas of guanethidine and its derivatives guanidine and azacyclooctane. All experiments were performed with three biological replicates.

The guanethidine (I) molecule primarily consists of a guanidine group and an azacyclic octane group [30]. To gain further insights into the specific moieties of guanethidine that enhance the activity of tetracycline against resistant bacteria, we assessed the efficacy of guanidine (II), a derivative compound of the guanidine group, in combination with tetracycline, as well as azacyclooctane (III), a derivative compound of the azacyclic octane group, in combination with tetracycline. The potency of the synergistic effect was assessed based on the fold decrease in MIC and the FICI value. Briefly, guanidine (II) exhibited synergistic activity (FICI ≤ 0.5) when used alone, with a 16-fold enhancement in MIC, while azacyclooctane (III) only showed an additive effect (0.5 < FICI ≤ 1.0) without any synergism (Figure 13B,C). Interestingly, when guanidine (II) was added at a sub-inhibitory concentration, the synergistic effect of guanethidine (I) and tetracycline showed little difference compared to the blank control group; however, when azacyclooctane (III) was added at a sub-inhibitory concentration, the synergistic effect was significantly enhanced (Figure 13A,D,E).

### 2.9. The Combination Did Not Increase the Toxicity of In Vitro Therapy

**Figure 14:**
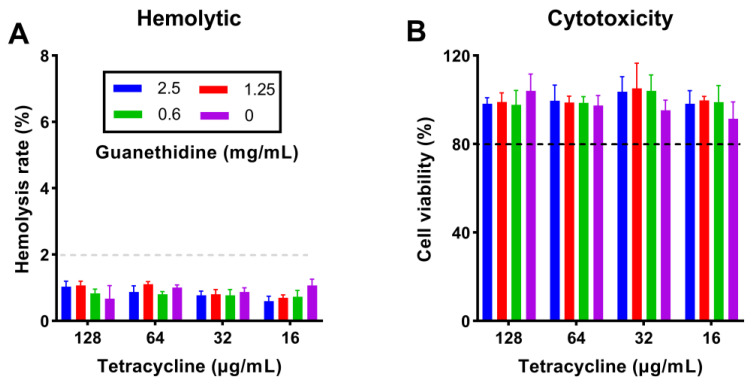
(**A**) The cytotoxicity of different concentrations of guanethidine in combination with tetracycline, and (**B**) the hemolysis of different concentrations of guanethidine in combination with tetracycline. (**A**) and (**B**) are illustrated using the same colour markings. All experiments were performed with three biological replicates.

The critical question in clinical combined therapy is whether the toxicity of combining antibiotics and adjuvants increases [9]. We measured the hemolytic and cytotoxic effects of guanethidine combined with tetracycline on mammalian cells. The co-administration of the two drugs at high doses did not result in any significant impact on rabbit red blood cell (RBC) hemolysis or RAW 264.7 cytotoxicity (Figure 14).

### 2.10. The Combination Did Not Alter Blood Routine or Biochemical Parameters in Healthy Mouse Models

The safety of combined drugs is crucial. In vitro research indicates that the combination of guanethidine and tetracycline is generally well-tolerated. To further investigate, we assessed whether this combination affects the basic vital signs of healthy mouse models. The concurrent administration of guanethidine and tetracycline did not result in any significant changes to the blood routine or blood biochemical parameters in healthy mouse models (Tables S2 and S3).

### 2.11. The Combination Enhanced the Survival Rate of Animals Infected with Multidrug-Resistant E. coli C3

**Figure 15:**
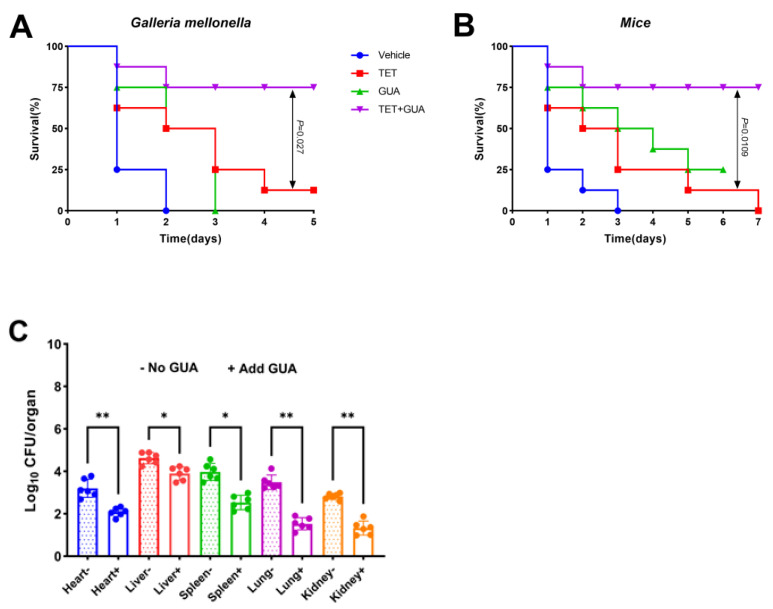
(**A**) The survival rate of Galleria mellonella larvae infected with *E. coli* C3 (10^6^CFU/mL). Each group was treated with tetracycline (35 mg/kg) or guanethidine (10 mg/kg) alone or in combination (35 mg/kg +10 mg/kg) (*n* = 8 per group). (**B**) The survival of mice infected with *E. coli* C3 (10^8^CFU/mL). Each group was treated with tetracycline (35 mg/kg) or guanethidine (10 mg/kg) alone or in combination (35 mg/kg +10 mg/kg) (*n* = 8 per group). *p* values were tested using the Mantel–Cox test, with *p* < 0.05 indicating a significant change. (**C**) *E. coli* C3 (10^6^ CFU/mL) infected mice were treated with tetracycline (35 mg/kg) or guanidine combined with tetracycline (35 mg/kg + 10 mg/kg) to determine the bacterial load in the heart, liver, spleen, lung, and kidney (*n* = 6 per group). In the *t*-test, * indicates *p* < 0.05, a significant difference, and ** indicates *p* < 0.01, indicating a highly significant difference.

We assessed whether this synergy would result in a positive outcome in an animal model of infection. The combination of guanethidine and tetracycline (Figure 15A) significantly enhanced the survival rate in mouse infection models compared to those treated with tetracycline alone (*p* < 0.05). The Galleria mellonella larval infection model yielded similar outcomes (Figure 15B). Furthermore, the combination of guanethidine and tetracycline was tested on the major organs (heart, liver, spleen, lung, and kidney) in an infected mouse model. The bacterial load of mice treated with guanethidine combined with tetracycline was significantly lower than that of those treated with tetracycline alone, and the effect of guanethidine combined with tetracycline on the bacterial load of heart, lung, and kidney reached a very significant level (Figure 15C).

## 3. Discussion

The antibiotic resistance of pathogenic bacteria is increasingly severe, but the lack of new therapeutic drugs is even worse [31]. Antibiotic adjuvants are a promising strategy because of their relatively low cost and short time consumption [32]. Given that discovering antibiotic adjuvants from unknown compounds may result in increased time or economic cost, we screened for novel tetracycline antibiotic adjuvants from PADs, as these compounds have clearly established toxicological and pharmacological profiles. Previously, the researchers discovered six non-antibacterial drugs such as benserazide from 1057 PADs [33]. These drugs demonstrated synergistic effects with minocycline against resistant Pseudomonas aeruginosa. The screening library involved the drug list of the World Health Organization and the US Food and Drug Administration. Additionally, statins were found to restore the sensitivity of methicillin-resistant Staphylococcus aureus to penicillin treatment [34]. Auranofin showed potential in enhancing the effectiveness of carbapenem drugs and polymyxin against multidrug-resistant bacteria [35]. These examples could inspire us to explore new tetracycline antibiotic adjuvants from PADs. We demonstrated that guanethidine greatly enhanced the inhibitory effect of most tetracyclines (except tigecycline) on strains carrying the gene encoding the TetA efflux pump. It is possible that *tetA* only mediates low levels of tigecycline resistance [36]. Guanethidine remains effective even in cell culture medium that simulates the physiological environment of animals. We also found that the combination of guanethidine and tetracycline significantly inhibited the development of resistance, with no resistant mutants appearing in the combination group compared with tetracycline alone, whereas the tetracycline alone group produced highly resistant strains with a 32- to 64-fold increase in MIC.

*E. coli*, a Gram-negative bacterium with an impermeable outer membrane, hinders the effective penetration of many antibiotics [37]. Interestingly, guanethidine can disrupt the outer membrane integrity of *E. coli*. We hypothesize that the hydrogen bonds between phosphate groups on the cell membrane and the guanidine group of guanethidine may be the cause of the impaired cell membrane function. The fluorescence intensity data of fluorescent dyes NPN, PI, and DISC_3_(5) support this speculation. This is similar to a guanidine polymer antibiotic adjuvant [38]. Additionally, the impact of guanethidine on proton motive force may affect the function of the TetA efflux pump driven by proton motive force. The mechanism is similar to lopatadine (an adjuvant of minocycline) [33], or metformin (an adjuvant of doxycycline) [9].

Our study showed that guanethidine combined with tetracycline only had a synergistic effect on *tetA*-positive strains, but almost no synergistic effect on *tetA*-negative strains. We speculate that guanethidine mainly acts on *tetA*. To further investigate, the constructed plasmid containing *tetA* was transformed into competent *E. coli DH5α* cells, and from the transformed cells, a tetracycline-resistant strain named *E. coli DH5α-ZMN1* carrying the *tetA* gene was selected. We found that guanethidine exhibited a synergistic effect when combined with tetracycline or doxycycline against *E. coli DH5α-ZMN1*, resulting in a reduction of the MICs by up to 16-fold. The molecular docking results indicate that guanethidine can stably bind to the binding site of the efflux pump protein TetA. This is because guanethidine contains a guanidine group with multiple hydrogen bond donors that can form multiple hydrogen bonds with acidic amino acid residues in the binding site of the protein. The average hydrogen bond distance is 1.73 Å, which is shorter than the typical range of 2.2 to 3.6 Å for conventional hydrogen bonds. In conclusion, these mechanisms contribute to the accumulation of tetracycline antibiotics within bacterial cells to overcome resistance mediated by TetA.

Guanethidine is a small molecular compound with a simple molecular structure, primarily composed of a guanidine group and azacyclic octane groups [23,39,40]. Our findings indicate that the guanidine group plays a crucial role in the synergism with tetracycline, while the azacyclic octane group enhances the synergistic effect. As a basic group, the guanidine group is capable of forming hydrogen bonds and inducing electrostatic reactions, which are critical to the antibacterial activity of these compounds. On the other hand, the presence of a hydrophobic cycloalkyl azacyclic octane group enhances the compound’s lipid solubility, which may affect its bioavailability and cellular permeability.

Finally, guanethidine combined with tetracycline has favorable safety and efficacy, which indicates that guanethidine is a potential tetracycline adjuvant and can be used to treat clinically related pathogenic bacteria. The discovery of guanidine compounds such as guanethidine encourages the search for more candidate drugs with synergistic effects that could serve as potential antibiotic adjuvants, particularly in exploring new antibacterial adjuvants from previously marketed drugs. However, more extensive clinical trials are essential to validate the potential efficacy of guanethidine as an adjuvant to tetracycline antibiotics.

## 4. Materials and Methods

### 4.1. Bacteria and Reagents

The strains and cells utilized in this study came from the pharmacological Laboratory of Jilin Agricultural University. All strains were grown in Mueller–Hinton broth (MHB, Qingdao Hopebio, Qingdao, China). RAW264.7 cells were grown in Dulbecco’s Modified Eagle’s Medium (DMEM, TransGen Biotech, Beijing, China) supplemented with 10% heat inactivated fetal bovine serum (FBS, Gibco, Shanghai, China). Drugs were obtained from Macklin (Shanghai, China).

### 4.2. MIC Measurements

The CLSI 2020 guideline was followed by determining MICs for all compounds using the standard broth microdilution method [41]. Select a single colony of *E. coli* C3 and inoculate it into Mueller–Hinton broth (MHB) medium, then culture it on a shaker at 37 °C (200 rpm) until it reaches the logarithmic growth phase, and adjust the bacterial concentration to 10^6^ CFU/mL. Drugs were 2-fold diluted in a 96-well microtiter plate, before being topped off with a 10^6^ CFU/mL bacterial solution in equal proportions. Incubation at 37 °C for 12 to 16 h. The MIC values were defined as the lowest concentrations of antibiotics with no visible growth of bacteria.

### 4.3. FIC Index Determination

Checkerboard assays were used to measure synergistic activity between compounds and antibiotics and the FICI [42]. Antibacterial drugs were 2-fold diluted along the abscissa while compounds were 2-fold diluted along the ordinate. Each well received a mixture of 10^6^ CFU/mL bacterial solution and was incubated at 37 °C for 12 to 16 h. The optical density of each well at 600 nm was determined by a Multiskan FC Microplate reader (Thermo, Waltham, MA, USA).

The FIC index (FICI) was determined by using the formula that follows [43].

FIC index = MIC_ab_/MIC_a_ + MIC_ba_/MIC_b_ = FIC_a_ + FIC_b_

where MIC_a_ is the MIC of compound A alone, MIC_ab_ is the MIC of compound A in combination with compound B, MIC_b_ is the MIC of compound B alone, MIC_ba_ is the MIC of compound B in combination with compound A, FIC_a_ is the FIC index of compound A, and FIC_b_ is the FICI of compound B. A synergistic effect is defined as an FICI of ≤0.5, an additive effect is defined as an FICI of >0.5 and ≤1.0, no effect is defined as an FICI of ≥1.0 and <4.0, and an antagonistic effect is defined as an FICI of ≥4.0 [41].

In addition, other reagents or drugs can be added as needed, or the pH of the culture medium can be adjusted.

### 4.4. Determination of Growth Curve and Time–Kill Curve

The strains at 0.5 McFarland were diluted 1:100, incubated with different concentrations of drugs, and monitored for absorbance at different times using a microplate reader. On the other hand, a continuous dilution of bacterial liquid was used for colony counting [44].

### 4.5. Resistance Development Studies

*E. coli* ATCC 25922 (0.5 McFarland) was diluted 1000-fold during the exponential growth phase into fresh MHB medium supplemented with either antibiotics (tetracycline, doxycycline, minocycline) at sub-inhibitory concentrations (0.25× MIC) or antibiotics plus 0.25-fold MIC of guanethidine (1.25 mg/mL). The MIC of the cultures was determined after 24 h of incubation at 37°C. At the same time, such cultures were diluted in the adjusted 0.25-fold MIC drug for the next round of passaging. This process was repeated for 30 days and the fold increase in antibiotic MIC relative to the initial MIC was calculated [9].

### 4.6. Fluorescence Assay

In the fluorescence assay, *E. coli* C3 was used as an indicator bacterium. All bacterial preparations used in the measurements were processed in a similar manner. In brief, the bacteria were cultured overnight at 37 °C at 200 rpm. The culture was then washed and cleaned with HEPES II (5 mM) or PBS (10 mM) three times. The bacterial suspension’s OD600 was standardized to 0.5 in the same buffer, and a fluorescent dye was added. The bacteria were incubated at 37 °C for 30 min, after which 100 µL of probe-labeled bacterial cells were added to 96-well plates and 100 µL of guanidine hydrochloride (final concentration of 0 to2.5 mg/mL) was added. The fluorescence intensity was measured on a fluorescence microplate reader (Molecular Devices, San Jose, CA, USA) after incubation for 30 min.

#### 4.6.1. Outer Membrane Permeability Assay

HEPES washing solution and an NPN (10 μM) fluorescence probe were utilized. The fluorescence of culture content was measured with an excitation wavelength of 350 nm and an emission wavelength of 420 nm. The positive control was 10 mM colistin [45].

#### 4.6.2. Inner Membrane Permeability Assay

PBS washing solution and a PI (10 nM) fluorescence probe were utilized [11]. The fluorescence of culture content was measured with an excitation wavelength of 535 nm and an emission wavelength of 615 nm. The positive control was 1% Triton X-100.

#### 4.6.3. Membrane Potential Gradient Assay

HEPES was used for washing and DiSC_3_(5) (0.5 μM) was used for fluorescence. The fluorescence of culture content was measured with an excitation wavelength of 622 nm and an emission wavelength of 670 nm [46].

#### 4.6.4. Proton Motive Force Assay

HEPES washing solution and a BCECF-AM (20 μM) fluorescence probe were utilized. To measure the fluorescence of culture content, a wavelength was used that was 488 nm excitation and 522 nm emission [46].

#### 4.6.5. Total ROS Measurement

HEPES washing solution and a DCFH-DA (10 μM) fluorescence probe were utilized [42]. To measure the fluorescence of culture content, a wavelength was used that was 488 nm excitation and 525 nm emission.

### 4.7. ATP Determination

Intracellular ATP levels of the strains were determined using an ATP Assay Kit (Jiancheng, Nanjing, China) [47]. A single colony of *E. coli* C3 was placed in MHB culture medium and shaken overnight at 37 °C (200 prm). After washing the bacterial suspension with PBS three times, the cell density was adjusted to an OD600 of 0.5. Different concentrations of guanidine (0–2.5 mg/mL) were added and incubated for 1 h. The mixture was then centrifuged at 4 °C (8000 rpm, 20 min) to collect the supernatant. The supernatant was placed in ATP working solution and mixed for 5 min. The luminescence was measured by a Tecan infinite M200 Pro Microplate Reader. Then, the value was input into the formula to calculate the ATP concentration.

### 4.8. Construct and Transform the Plasmid Containing the tetA Gene

#### 4.8.1. The *tetA* Gene Was Amplified

The genome of *E. coli* C3 (with the *tetA* gene) was extracted (Ezup columnar bacterial genomic DNA extraction kit, Sangon, Shanghai, China) after being cultured overnight. The following amplification conditions were used for *tetA* gene amplification by PCR: The optimized primers were as follows (*for*: GAGGATCCCCGGGTACTCAGCGATCGGCTCGTTG and *rev*: GAGGAATTCACCATGGTGAAACCCAACAGACCCCTG) [48]. The amplification product length was 1200 bp. DNA Polymerase was a PrimeSTAR Max DNA Polymerase enzyme. The reaction conditions were as follows: predenaturation at 98 °C for 3 min; denaturation at 98 °C for 10 s; annealing at 55 °C for 10 s; and elongation at 72 °C for 15 s. The reaction was extended at 72 °C for 5 min.

#### 4.8.2. The Amplification of the *pBAD* Plasmid

The conditions are as follows:

The optimized primers were as follows:

(*for*: CATGGTGAATTCCTCCTGCTAGC and *rev*: GTACCCGGGGATCCTCTAGAG) [49]. The amplified length was 4542 bp; DNA Polymerase was a PrimeSTAR Max DNA Polymerase enzyme; the reaction conditions were pre-denaturation at 98 °C for 3 min, denaturation at 98 °C for 10 s, annealing at 55 °C for 10 s, and extension at 72 °C for 50 s. After 72 °C, the reaction was extended for 5 min.

#### 4.8.3. Construction and Transformation of Plasmids

The amplified product was verified by gel electrophoresis and purified by a gel recovery kit (Sigma-Aldrich, St. Louis, MO, USA). The purified *tetA* gene and *pBAD* plasmid product were seamlessly linked by Infusion enzyme (Takara, San Jose, CA, USA) (50 °C, 15 min), and the ligated product was transferred into *E. coli* DH5α competent state by heat shock transformation. The specific process was as follows: 5 μL of the ligation product was added to 120 μL of competent cells in an ice-water mixture, and incubated at 4 °C for 30 min. The mixture was then heated at 42 °C for 90 s, quickly removed from the ice bath for 5 min, mixed with 1 mL of culture medium, placed in a shaker for 2 h for amplification, and then removed and centrifuged at 8000 rpm for 2 min to retain the bacteria. The bacteria were mixed with the remaining culture medium in the tube and spread on an ampicillin-resistant agar plate containing 100 μg/mL ampicillin, incubated at 37 °C for 20 h, and single colonies were picked up and cultured to the logarithmic growth phase on MHB medium. PCR amplification of the gene *tetA* was performed, and Sanger sequencing was used for verification. At the same time, the plasmid was extracted from the transfected bacterial strain using the SanPrep Column Plasmid Mini-Preps Kit, (Sangon, China) and Sanger sequencing for verification [50].

### 4.9. Molecular Docking

The 3D structure of guanethidine was obtained from the PubChem database. The structure of the protein TetA was constructed using the online server Swiss-Model. Molecular docking was performed by MOE [51,52,53].

### 4.10. RT-PCR Analysis

A single colony of *E. coli* C3 was placed into MHB liquid culture medium and shaken overnight. Then, the bacterial suspension and culture medium were diluted at a ratio of 1:100 and transferred to 1.5 mL centrifuge tubes. Different concentrations of guanethidine (0–2.5 mg/mL) were added and incubated at 37 °C until the logarithmic phase (OD600 = 0.5). RNA was extracted using the EASY spin Plus kit (Aidlab, Hong Kong, China), followed by reverse transcription into cDNA utilizing the PrimeScript RT reagent Kit with gDNA Eraser (Takara, Beijing, China). Finally, *tetA* gene expression was quantified through real-time fluorescence-based quantitative PCR. The TetA gene primer is as follows (*for*: TTTCGGGTTCGGGATGGT and *rev*: GGAAACAGCCCGTCAGGAA) [9]. Primers for the reference gene *rsmC* were as follows (*for*: GAAATTCTGGGCGAATACA and *rev*: CTTTCACCTCGGAAAAGACG). The reaction system was 20 μL: Light Cycler 480 SYBR Green I Master (2×) 10 μL, ddH_2_O 8 μL, cDNA 1 μL, and upstream and downstream primers 0.5 μL each. The reaction procedure was predenaturation at 95 °C for 5 min followed by denaturation at 95 °C for 10 s. Each experiment was repeated three times with 40 cycles of annealing at 60 °C for 15 s and extension at 72 °C for 20 s. Roche Lightcycler 480 II software (Version 1.5) was used for data analysis. The fold change in gene expression was calculated by the 2^−ΔΔCt^ method.

### 4.11. Gene tetA Integrity Analysis

*E. coli* C3 was cultured in MHB containing sub-inhibitory concentrations of guanethidine (2.5 mg/mL) at 37 °C for 24 h, followed by the measurement of the MIC. Cultivation was conducted for 7 consecutive days, with each day’s culture environment consisting of guanethidine at the sub-inhibitory concentration established in the previous day’s generation. Using the same methodology, an equal volume of PBS was cultured continuously for 7 days as a control group. Then the genome was extracted with the MiniBEST Universal Genomic DNA Extraction Kit (TaKaRa, China), and the *tetA* gene was amplified by PCR. The primers were as follows: (*for*: TACGCCGACCTCGTTCAA and *rev*: TTTCCTTACTGGGCTTTCTCA). The length of the amplified fragment was 1581 bp. The total reaction system was 25 μL: ddH_2_O 8.5 μL, 2xTaq PCR MasterMix 12.5 μL, template 2 μL, and primer 1 μL, respectively. The reaction conditions of PCR were pre-denaturation at 94 °C for 5 min, denaturation at 94 °C for 30 s, annealing at 60 °C for 30 s, extension at 72 °C for 1 min for 40 s, 30 cycles, and extension at 72 °C for 10 min [54].

### 4.12. Antibiotics Accumulation Analysis

*E. coli* C3 and *E. coli* ATCC25922 were cultivated to the logarithmic growth phase. Bacterial cells were collected by centrifugation at 4 °C, washed three times with PBS, and resuspended in PBS with a concentration of 10^10^ CFU/mL, followed by equal distribution. Solutions containing different concentrations of guanethidine with tetracycline were mixed, and incubated at 37 °C for 15 min. The sample was centrifuged at 13,000× *g* for 5 min at 4 °C, followed by two washes with PBS to remove unbound material, and the bacterial cells were retained. The treated bacterial cells were resuspended in 400 μL of sterile water, followed by sonication and centrifugation at 13,000× *g* for 2 min to collect the supernatant. The remaining bacterial cells were resuspended in 200 μL of methanol, vortexed, and then centrifuged to collect the supernatant. After merging the supernatants from two centrifugations at 13,000× *g* for 20 min, collect the supernatant for further analysis. The quantification of intracellular antibiotic accumulation was conducted using LC-MS/MS [9].

### 4.13. Measurement of Hemolysis Activity

A 2% rabbit red blood cell suspension was incubated with the drug at 37 °C for 1 h, then centrifuged at 1000× *g* for 10 min. The supernatant from the previous step was transferred to a 96-well plate, and the absorbance was measured at 576 nm. The negative control group consisted of red blood cells mixed with normal saline, and the positive control group consisted of red blood cells mixed with 0.2% Triton X. The hemolysis rate was calculated as the difference between the absorbance of the sample and the negative control, and the ratio of this difference to the difference between the absorbance of the positive control and the negative control [55].

### 4.14. Cytotoxicity Assays

The concentration of RAW mouse macrophages was adjusted to 1.5 × 10^5^ cells/mL after they were cultured to the desired density. A total of 100 μL of the cell suspension was dispensed and added to 96-well plates for cell culture. The cells were cultured for 6 h to stabilize, and then 100 μL of guanethidine and tetracycline with different concentrations were added for incubation for 12 h. The cell survival rate was detected by the Cell Counting Kit-8 (beyotime, Shanghai, China). The cell survival rate was calculated as the ratio of the difference between the absorbance of the sample and the negative control to the difference between the absorbance of the positive control and the negative control [55].

### 4.15. In Vivo Toxicity Test

The mice were randomly allocated into three groups. The first group served as a blank control, receiving an intraperitoneal injection of 0.9% normal saline. In the second group, guanethidine (10 mg/kg) and tetracycline (35 mg/kg) were administered intraperitoneally simultaneously, and blood was collected 2 d later. In the third group, blood was collected 7 d after the administration of guanethidine (10 mg/kg) and tetracycline (35 mg/kg). Blood was taken from the eyeball and placed in a heparin sodium anticoagulation tube. The corresponding indexes were detected by an automatic blood cell analyzer (Mindray, China) and automatic biochemical analyzer (Mindray, Shenzhen, China) [38].

### 4.16. Galleria Mellonella Infection Mode

Galleria mellonella larvae were randomly divided into 4 groups, with 8 larvae in each group. A total of 10 μL *E. coli* C3 suspension (10^6^ CFU/mL) was injected into the right posterior abdomen of the larvae, and 10 μL guanethidine (10 mg/kg), tetracycline (35 mg/kg), and guanethidine (10 mg/kg) and tetracycline (35 mg/kg) were injected into the left abdomen of the larvae one hour after infection. The positive control was injected with the same amount of carrier solvent, and the treated larvae were placed in an incubator, and the survival of larvae within 5 d was observed and recorded [56].

### 4.17. Mouse Intraperitoneal Infection Model

Mice were randomly divided into 4 groups, each consisting of 8 mice, with an equal number of males and females. They were fed for 7 d in a closed environment to adapt to it. Mice were intraperitoneally injected with *E. coli* C3 suspension (bacterial solution concentration 10^8^ CFU/mL), and the drugs were intraperitoneally injected 1 h later. The injection dose was the same as in the Galleria mellonella larval infection model. The positive control was injected with the same amount of vehicle solvent. The survival status of mice was observed and recorded within 7 d [56]. The mice were raised in strict accordance with the ‘Regulations for the Management of Laboratory Animals’ approved by the State Council of China (2017), and the study was approved by the Animal Ethics and Welfare Committee of Jilin University. The laboratory animal use license number is JNK20230501-1.

### 4.18. Determination of Bacterial Load in Organs

The mice were randomly divided into 2 groups, 6 mice in each group, half male and half female, and were kept under closed conditions for 7 d to adapt to the environment. Mice were intraperitoneally injected with *E. coli* C3 suspension (the concentration of bacterial solution was 10^6^ CFU/mL). After 1 h, one group of mice was intraperitoneally injected with tetracycline 35 mg/kg. The other group received an intraperitoneal injection of guanidine 10 mg/kg and tetracycline 35 mg/kg. After 24 h of infection, the heart, liver, spleen, lungs, and kidneys were dissected under sterile conditions. The tissues were homogenized and serially diluted, followed by spreading onto MacConkey agar culture medium containing doxycycline resistance. The plates were then incubated at 37 °C for 20 h, and the bacterial colonies were counted [44].

### 4.19. Statistical Analysis

Statistical analysis was performed using an unpaired *t*-test between two groups and one-way ANOVA among multiple groups. SPSS22.0 was used for statistical analysis. Quantitative data were expressed as mean ± standard deviation, and *p*-values were considered statistically significant (* *p* <0.05, ** *p* < 0.01, and *** *p* < 0.001; ns represents insignificant).

## 5. Conclusions

In this study, we found that guanethidine restored the susceptibility of multidrug-resistant *E. coli* with the *tetA* gene to tetracyclines and enhanced the antibacterial efficacy of tetracycline antibiotics. Guanethidine also delayed the development of resistance to tetracyclines in bacteria effectively. Notably, the synergistic effect was not observed in *E. coli* strains without the *tetA* gene. The main mechanism of guanethidine is to inhibit the efflux pump of TetA and effectively increase the concentration of intracellular tetracycline. Additionally, guanethidine can also affect the integrity of the outer membrane of bacterial cells, thereby interfering with the PMF associated with the cell membrane, as well as intracellular metabolic activities such as the increase in reactive oxygen and the decrease in ATP levels, further weakening the bacteria’s ability to survive. It is worth noting that the synergistic effects of guanethidine are closely associated with its pharmacophoric guanidine group. As for safety and efficacy in vivo, guanethidine combined with tetracycline did not show significant toxicity in healthy mouse models, and significantly improved the survival rate of infected animal models, and significantly reduced the bacterial load in major organs. These results indicate that guanethidine can enhance the antibacterial activity of tetracycline antibiotics both in vivo and in vitro, and the combination therapy is safe. Therefore, guanethidine shows the potential to be a novel tetracycline antibiotic adjuvant, and further research can be conducted to further develop and optimize it.

## Data Availability

The original contributions presented in the study are included in the article/Supplementary Materials; further inquiries can be directed to the corresponding authors.

## Supplementary Materials

The following supporting information can be downloaded at: https://www.mdpi.com/article/10.3390/antibiotics13100973/s1, Table S1: *E. coli* C3 resistance profile. Table S2: Complete blood count (CBC) in healthy mouse models. Table S3: Blood biochemistry analysis in healthy mouse models.
