# Supplementary material for: Guanethidine Restores Tetracycline Sensitivity in Multidrug-Resistant Escherichia coli Carrying tetA Gene
Source: Antibiotics (Basel). 2024 Oct 15;13(10):973. doi: 10.3390/antibiotics13100973 (PMC11504368; doi:10.3390/antibiotics13100973)
Supplement: Supplementary file 1 [file antibiotics-13-00973-s001.zip › antibiotics-3191168-supplementary.pdf]

Supplementary Material

Guanethidine Restores Tetracycline Sensitivity in Multidrug-Resistant *Escherichia coli* Carrying *tetA* Gene

Xiaoou Zhao [1,2], Mengna Zhang [1,3], Zhendu Zhang [2], Lei Wang [2], Yu Wang [1,4], Lizai Liu [2], Duojia Wang [2], Xin Zhang [2], Luobing Zhao [2], Yunhui Zhao [2], Xiangshu Jin [2], Xiaoxiao Liu [2,*] and Hongxia Ma [1,5,*]

1 College of Animal Science and Technology, Jilin Agricultural University, Xincheng Street No. 2888, Changchun 130118, China; zhaoxo4466@163.com (X.Z.); zhangmn2021@126.com (M.Z.); wang2311472521@163.com (Y.W.)
2 Institute of Animal Husbandry and Veterinary Medicine, Jilin Academy of Agricultural Science, Kemao Street No. 186, Gongzhuling 136100, China; zhangzhendu@126.com (Z.Z.); florawong1982@126.com (L.W.); 15662165476@163.com (L.L.); 15104487977@163.com (D.W.); 16624441717@163.com (X.Z.); a13630969537@163.com (L.Z.); yunhui0328@126.com (Y.Z.); 13894469000@163.com (X.J.)
3 College of Veterinary Medicine, Northwest A&F University, Xinong Street No. 22, Yangling 712100, China
4 College of Life Sciences, Jilin Normal University, Haifeng Street No. 1301, Siping 136000, China
5 The Engineering Research Center of Bioreactor and Pharmaceutical Development, Ministry of Education, Jilin Agricultural University, Xincheng Street No.2888, Changchun 130118, China
* Correspondence: xiaoxiaoliu123456@163.com (X.L.); hongxia0731001@163.com (H.M.)

Table S1 *E. coli* C3 resistance profile.

Antimicrobial drugs	MIC(μg/mL)
Chlortetracycline	128
Oxytetracycline	128
Tetracycline	128
Doxycycline	32
Minocycline	16
Tigecycline	0.5
Ampicillin	512
Ceftriaxone	256
Vancomycin	16
Polymyxin B	0.5
Rifampicin	128
Enrofloxacin	256
Azithromycin	2
Florfenicol	256
Gentamicin	16
Sulfamonomethoxine Sodium	512

Table S2 Complete blood count (CBC) in healthy mouse models.

Test Items	Blank Control (n=6)	2 Days after Drug Combination (n=6)	7 Days after Drug Combination (n=6)
White Blood Cells (per 10^9/L)	2.670±0.195	2.72±0.151(ns)	2.705±0.253(ns)
Neutrophil Percentage (%)	2.568±0.150	2.43±0.342(ns)	2.545±0.307(ns)
Lymphocyte Percentage (%)	74.748±3.673	73.098±2.417(ns)	72.510±4.016(ns)
Monocyte Percentage (%)	4.440±0.684	4.735±0.467(ns)	4.888±0.282(ns)
Eosinophil Percentage (%)	0.025±0.011	0.038±0.018(ns)	0.040±0.016(ns)
Basophil Percentage (%)	8.498±0.617	8.135±1.240(ns)	8.340±1.268(ns)
Neutrophils (per 10^9/L)	0.060±0.016	0.075±0.011(ns)	0.070±0.012(ns)
Lymphocytes (per 10^9/L)	2.418±0.543	2.800±0.617(ns)	2.800±0.617(ns)
Monocytes (per 10^9/L)	0.165±0.056	0.178±0.074(ns)	0.188±0.022(ns)
Eosinophils (per 10^9/L)	0.00578±0.003	0.003±0.002(ns)	0.004±0.001(ns)
Basophils (per 10^9/L)	0.253±0.035	0.260±0.051(ns)	0.298±0.049(ns)
Red Blood Cells (per 10^{12}/L)	8.925±0.497	9.185±0.315(ns)	8.678±0.677(ns)
Hemoglobin (g/L)	141.73±10.926	129.15±14.017(ns)	134.405±7.866(ns)
Hematocrit (%)	0.453±0.026	0.388±0.054(ns)	0.348±0.106(ns)
Mean Corpuscular Volume (fL)	52.780±2.687	56.945±1.927(ns)	56.160±1.763(ns)
Mean Corpuscular Hemoglobin (pg)	16.693±0.863	17.750±0.495(ns)	17.580±0.817(ns)
Mean Corpuscular Hemoglobin Concentration (g/L)	278.018±8.979	281.495±5.348(ns)	275.73±11.148(ns)
Red Blood Cell Distribution Width (%)	16.300±1.617	18.075±1.119(ns)	16.825±0.626(ns)
Platelets (per 10^9/L)	820.61812.122	834.033±9.776(ns)	843.85±30.402(ns)
Platelet Distribution Width (%)	11.375±0.934	9.950±0.555(ns)	10.000±0.930(ns)
Mean Platelet Volume (fL)	6.388±0.717	6.965±0.294(ns)	6.858±0.383(ns)
Plateletcrit (%)	0.538±0.076	0.560±0.130(ns)	0.485±0.129(ns)
Conventional C-Reactive Protein (mg/L)	<5	<5	<5
High-Sensitivity C-Reactive Protein (mg/L)	<3	<3	<3

Note: Data were analyzed using t-tests. * indicates $p<0.05$, representing a significant difference; ** indicates $p<0.01$, representing a highly significant difference; ns indicates $p>0.05$, representing no significant difference. n represents the sample size.

Table S3. Blood biochemistry analysis in healthy mouse models.

Test Items	Blank Control (n=6)	2 Days after Drug Combination (n=6)	7 Days after Drug Combination (n=6)
**Liver Indicators**			
Alanine Aminotransferase (ALT) (U/L)	57.825±2.599	61.025±3.937(ns)	60.7250±2.7114(ns)
Aspartate Aminotransferase (AST) (U/L)	160.300±3.764	159.300±4.906(ns)	160.275±9.8218(ns)
Albumin (g/L)	32.325±5.113	31.270±4.671(ns)	30.0250±3.9328(ns)
Total Protein (g/L)	57.650±5.293	59.100±2.865(ns)	57.3000±5.9720(ns)
**Kidney Indicators**			
Creatinine (μmol/L)	23.000±2.550	22.750±3.031(ns)	24.750±4.323(ns)
Urea Nitrogen (mmol/L)	7.500±1.118	7.500±0.500(ns)	7.250±0.829(ns)
Uric Acid (μmol/L)	127.750±3.767	128.750±3.767(ns)	120.250±6.057(ns)
**Metabolic Indicators**			
Total Cholesterol	2.675±0.311	2.575±0.653(ns)	2.650±0.658(ns)
Triglycerides	2.018±0.219	2.350±0.391(ns)	2.225±0.383(ns)
Blood Glucose	6.600±0.851	6.275±0.482(ns)	6.750±0.986(ns)

Note: Data were analyzed using t-tests. * indicates $p<0.05$, representing a significant difference; ** indicates $p<0.01$, representing a highly significant difference; ns indicates $p>0.05$, representing no significant difference. n represents the sample size.
